# Design and Validation of a Portable Machine Learning-Based Electronic Nose

**DOI:** 10.3390/s21113923

**Published:** 2021-06-07

**Authors:** Yixu Huang, Iyll-Joon Doh, Euiwon Bae

**Affiliations:** Applied Optics Laboratory, School of Mechanical Engineering, Purdue University, West Lafayette, IN 47907, USA; huang928@purdue.edu (Y.H.); idoh@purdue.edu (I.-J.D.)

**Keywords:** metal-oxide sensor, olfactory, portable instrument, food authentication, machine-learning, electronic nose

## Abstract

Volatile organic compounds (VOCs) are chemicals emitted by various groups, such as foods, bacteria, and plants. While there are specific pathways and biological features significantly related to such VOCs, detection of these is achieved mostly by human odor testing or high-end methods such as gas chromatography–mass spectrometry that can analyze the gaseous component. However, odor characterization can be quite helpful in the rapid classification of some samples in sufficient concentrations. Lower-cost metal-oxide gas sensors have the potential to allow the same type of detection with less training required. Here, we report a portable, battery-powered electronic nose system that utilizes multiple metal-oxide gas sensors and machine learning algorithms to detect and classify VOCs. An in-house circuit was designed with ten metal-oxide sensors and voltage dividers; an STM32 microcontroller was used for data acquisition with 12-bit analog-to-digital conversion. For classification of target samples, a supervised machine learning algorithm such as support vector machine (SVM) was applied to classify the VOCs based on the measurement results. The coefficient of variation (standard deviation divided by mean) of 8 of the 10 sensors stayed below 10%, indicating the excellent repeatability of these sensors. As a proof of concept, four different types of wine samples and three different oil samples were classified, and the training model reported 100% and 98% accuracy based on the confusion matrix analysis, respectively. When the trained model was challenged against new sets of data, sensitivity and specificity of 98.5% and 98.6% were achieved for the wine test and 96.3% and 93.3% for the oil test, respectively, when the SVM classifier was used. These results suggest that the metal-oxide sensors are suitable for usage in food authentication applications.

## 1. Introduction

Among the human senses, olfaction is a sensitive method involving the detection of small numbers of molecules in the environment. There are many applications of olfaction in everyday life; however, in many of these areas, such as food analysis, the use of odors has been limited since humans have several limitations when discriminating between different smells. For example, a high amount of experience and training is required, sensory results can be subjective rather than objective, and the nose is subject to sensory fatigue with time and has a limited ability to distinguish between very similar odors. Until relatively recently, there have been few electronic sensors sensitive or selective enough to replace operators trained to discriminate between different odors. Electronic noses have the ability to offer improvements in all of these aspects.

There are several types of sensors and instruments for sensing odors. Gas chromatography (GC) passes the sample through a small tube filled with a stationary phase in order to separate components by retention time [1]. This method can easily separate some compounds, while others produce overlapping peaks. Mass spectrometry (MS) also achieves high selectivity and, when combined with gas chromatography, is able to separate and identify most compounds. Despite the advantages of this technique (GC–MS), analysis is time-consuming and the equipment itself requires capital investment as well as extensive training to operate and interpret the results, all of which prevents its widespread deployment in portable and resource-limited environments [2,3]. Another type of sensor is the quartz crystal microbalance (QCM), which consists of a quartz crystal resonating at its resonant frequency [3,4,5,6]. When gas from a sample is passed over the QCM, the change in the resonant peak can be used to detect specific gases. Since the odors need only to stick to the coating, a wide variety of coatings can be used, meaning that this technique is sensitive to a large range of compounds. However, the circuitry to operate these sensors can be complicated, because the resonant frequencies are in the megahertz range and simultaneous detection of multiple types of gases requires a complicated manufacturing process [7]. Metal-oxide semiconductors are more commonly used than any other class of gas sensors to provide arrays for odor sensing [8]. This type of sensor detects gases by measuring the conductivity change due to the adsorption of gas molecules on the surface of a metal-oxide semiconductor [9]. Tin oxide is typically used as the sensitive layer. When heated, oxygen is adsorbed, changing the charge within the crystalline structure and thus changing the resistance. Reducing gases decrease the amount of oxygen in the layer, lowering the resistance [10]. Tin-oxide sensors have overlapping sensitivities and not very high selectivity, similarly to biological systems [4]. This is not a problem because the combination of output from an array of sensors can be used to determine the characteristic odors, and the sensor arrays can be tailored to a range of applications owing to the large number of varieties on the market. Moreover, for their simplicity, light weight, and relatively low cost compared to other sensing technologies, portable-type sensors that utilize metal-oxide detectors have already been reported in many applications [11].

Applications for a low-cost, widely available electronic nose include various food safety applications such as detecting food adulteration [12], food spoilage [13], or drugs [14]. Qi et al. reported a liquor discrimination sensor that can distinguish fake and real liquors [12]. While interesting, this sensor does not provide a concentration limit, requires a pump, must be washed out after every sample, and can report only binary classification results with four sensors. A series of TGS sensors have been used to differentiate cannabis samples; the system is still computer-controlled, with a large footprint, and also requires a pump to circulate the cannabis samples [14]. Other e-noses that have been reported commonly use a benchtop system with wall power and require a pump (or motor) and valves to operate the system [10,15,16]. Amari et al. reported using metal-oxide sensors to classify the age of raw milk using principal component analysis (PCA) and support vector machines (SVM) [17]. This design employed nitrogen to sample the headspace of each sample. Chen et al. used metal-oxide sensors to estimate the concentration of gases, but had the entire apparatus in a temperature- and humidity-controlled chamber [18]. Xu et al. used metal-oxide sensors to classify pecans; a purge of the system was required between samples [19]. Li et al. sampled the headspace of an enclosed container using multiple sensors with temperature cycling to obtain more data [20]. A portable power pack was also an option for running their electronic nose. Most sampling methods used an evaporation chamber and air flow from a pump, which increased the total system complexity and cost. It has been known for some time that bacteria emit different odors that can be used as biomarkers. This has been extensively explored with techniques such as GC–MS. Although applications of electronic nose technology have been limited by the low dimensionality of traditional metal-oxide sensor arrays, practical results have been achieved for the classification of a small number of bacterial and fungal species [21,22,23,24,25,26].

Several main techniques are used to classify different odors once data have been collected. The most common technique for electronic noses is principal component analysis. PCA has the ability to reduce high-dimensional data to much lower-dimensional data, significantly simplifying further analysis. Each principal component is orthogonal to other components and contains a fraction of the variance found in the original dataset. While PCA is useful for reducing the dimensionality of the data, it cannot be used to classify data on its own. For this task, support vector machines or neural networks, similar to biological systems, are used [7]. An SVM attempts to find a hyperplane that best separates the data [16]. K nearest neighbors (KNN) can also be used to classify odors.

Recognizing the growing interest in portable electronic noses and previously reported systems, this paper presents a portable metal-oxide sensor-based electronic nose that consists of ten different metal-oxide sensors. The battery-powered system has a fan to control the air flow, and the overall size fits within an adult’s palm, making it suitable for field-deployable applications. For validation of the proposed device, four wine and three oil samples were tested and the classification performance was compared among 24 types of machine learning classifiers.

## 2. Materials and Methods

### 2.1. Sensor Selection

**Figure 1:**
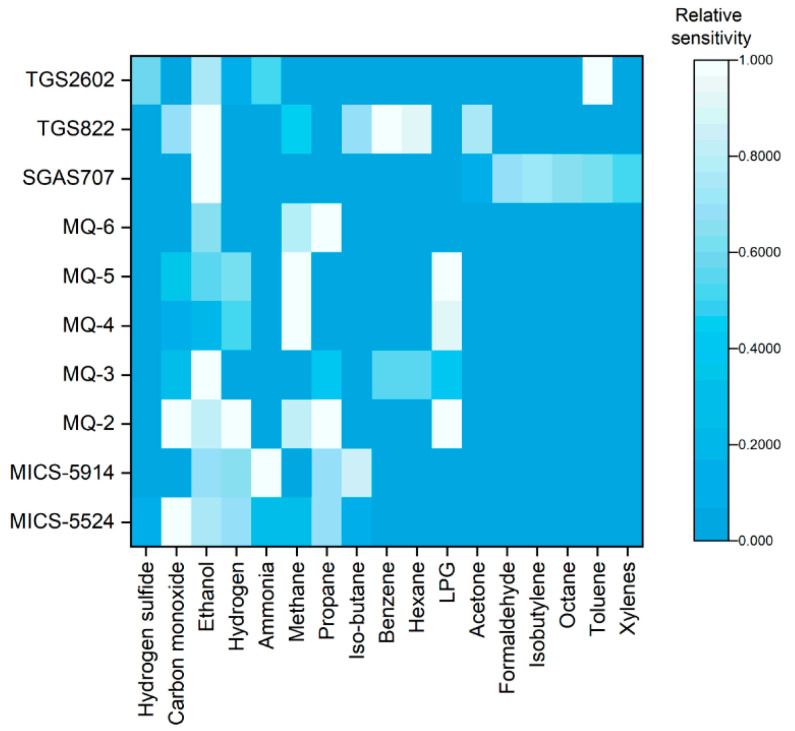
Comparison of relative sensitivity of 10 candidate metal-oxide sensors with respect to the detectability of 17 chemical species. Sensor selection was determined based on wider selection of chemical species with strongest sensitivities.

Since the goal was to build a low-cost, portable electronic nose, metal-oxide sensors were chosen. These sensors have small footprints, have low power consumption, and still provide high sensitivity for certain VOCs. Since VOCs are compounds that indicate the presence of certain types of biochemical activity, a matrix of potential sensors with their sensitivities was composed to assess the potential of the list of commercially available sensors (Figure 1). These data were obtained from the datasheets for each of the 10 sensors. Each sensor’s data were normalized based on the maximum sensitivity of each sensor, which was the slope of the concentration versus resistance change. In addition, they also showed the cross-sensitivities and response graphs of each sensor, allowing for more detailed responses for each sensor to be predicted. From this information, a list of sensors was selected in order to detect the maximum number of VOCs while limiting overlap between the sensors. The final set of sensors used were MQ-2 for flammable gases, MQ-5 for natural gas, MICS-5524 for carbon monoxide and natural gas, SGAS-707 for VOCs, MQ-3 for alcohol, MQ-4 for natural gas, MQ-6 for flammable gases, TGS-822 for VOCs, and TGS-2602 for air contaminants, based on Figure 1. Supplementary Materials Table S1 shows detailed information of the full list of surveyed sensors and the boldface row displays the selected sensors.

### 2.2. Circuit Design

The main controller was chosen to be STM32F031 (STMicroelectronics, Geneva, Switzerland). This CPU utilizes ARM Corex-M0 with 48 MHz frequency and operates on 3.3 V input. It also provides a 12-bit analog to digital converter (ADC) with input range of 0–3.6 V with several standard communication interfaces, such as universal asynchronous receiver transmitter (UART), serial port interface (SPI), and I2C protocol. Sensors chosen (see Section 2.1) were designed in a voltage divider arrangement from voltage common collector (VCC) of 5 V and ground. Metal-oxide sensors change their respective resistances, *R_S_*, as target VOCs adsorb to the sensor surface, and connecting to a precision resistor of *R*_0_ in series generates a voltage division as

$$V_{out}=\frac{1}{\frac{R_{s}}{R_{2}}+1}\;V_{CC}$$

where *V_out_* is the voltage read by the controller ADC channel. With this arrangement, an increase in gas concentration decreases *R_S_*, which in turn increases the voltage at the output so that a positive correlation between voltage and concentration is formed. In addition, the baseline voltage output for each sensor can be adjusted. Rather than designing an individual PCB board for each sensor, the sensor package was mounted in a 3D-printed case with custom-designed sockets for the sensors, and the breakout boards were soldered together. Potentiometers of various values were exposed in the top of the electronic nose in order to allow the baseline value for each sensor to be adjusted.

The programming and serial port of the microcontroller were exposed to allow for easy programming and data transfer. A fan (MC20080V1-000U-A99, DigiKey Electronics, Thief River Falls, MN, USA) was also included in order to draw the odors into the electronic nose.

**Figure 2:**
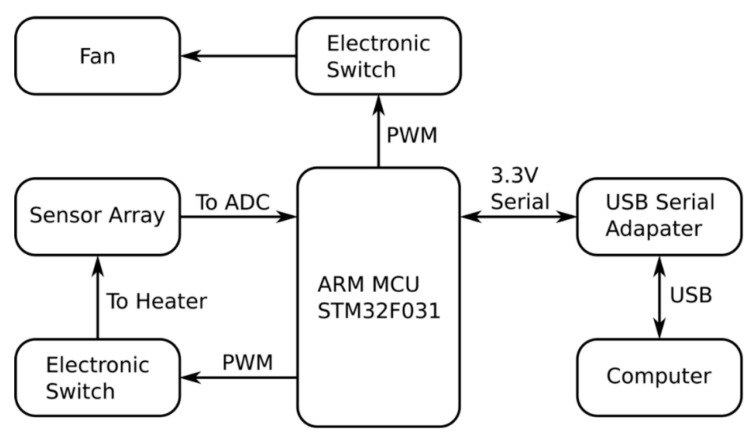
Functional block diagram of the e-nose. STM32F031 was used as the microcontroller unit that controls the fan and the heater by pulse width modulation (PWM). The 12-bit ADC receives the senso- array readings and transfers the data to the Raspberry Pi4 via a USB serial adapter.

**Figure 3:**
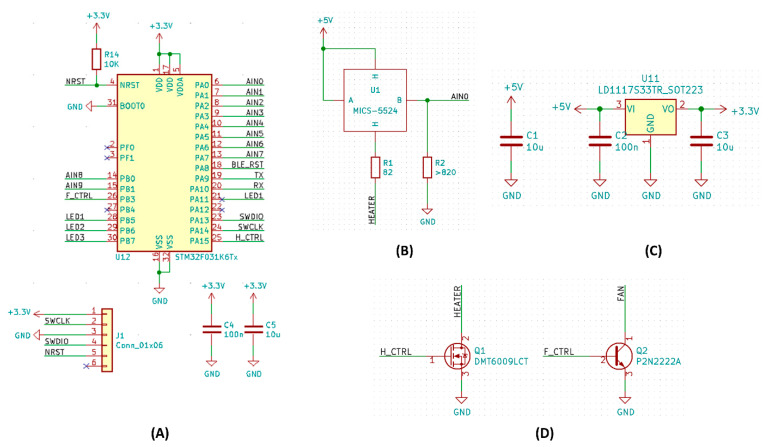
Circuit diagram of the proposed system. (**A**) STM32 microcontroller was used to control and collect all the data. (**B**) Each sensor is powered by a 5 V signal and enable pin, while a potentiometer (R2) is used to provide a voltage divider circuit. Shown here is the MICS-5524 sensor, which, unlike other sensors, requires a heater to operate. (**C**) The voltage regulator part is responsible for charging the battery. (**D**) The proportional control of heater and fan via transistors.

Two 2600 mAh, 3.7 V battery cells (18,650 size, Sparkfun Electronics, Niwot, CO, USA) were serially connected. A functional diagram of the overall system is shown in Figure 2, with the electronic wiring diagram provided in Figure 3 and Figure S1. The portable instrument developed was named electronic nose modules with machine learning algorithm (EMLA).

### 2.3. Data Acquisition

The sensors responded slowly, in the order of tens of seconds, to changes in the environment. Since the ADC of the microcontroller used was capable of one mega-sample per second, oversampling was used to increase the effective resolution of the sensors. For every *n* times the sensor was oversampled, assuming white noise, there was a $\sqrt{N}$ times increase in resolution and a decrease in noise [14]. Since the steady-state readings of the sensors were used for classification, only a low sampling rate was required. One sample per second was chosen as the sample rate. With 10 sensors, this allows for an oversampling rate of 5556×. With a 12-bit ADC, this gives approximately 18 bits of resolution from the STM32F0 series. The very simple software running on the microcontroller performed the oversampling and sent the data over the logic level serial port.

### 2.4. Calibration

To provide sensor selectivity and a quantitative response curve, calibration was performed with isopropyl alcohol. Then, 70% stock solution was diluted to 50, 35, and 15%, and approximately 100 µL of each solution was deposited on the 3D-printed sample tray. EMLA was turned on for 3 min, fan speed was set to the 16/255 level, and the heater to the maximum level (255/255). Once the baseline response was confirmed, 3D-printed sample tray was inserted under the fan. Data were collected for five minutes, recording one reading per second for all 10 sensors. Each sample challenge was repeated three times for statistical calculation. For plotting the calibration data, mean and standard deviation of the sensor responses for three replica were calculated and peak ADC output of each concentration for sensors that responded to isopropyl alcohol was plotted to check the response linearity.

### 2.5. Sample Preparation

For wine samples, four different wine samples were purchased from a local store: cabernet sauvignon (Central Valley, Chile, 2015), merlot (Columbia Valley, WA, USA, 2017), pinot noir (Sonoma County, CA, USA, 2018), and Zinfandel (Mendocino County, CA, USA, 2017). Each wine sample was kept in a refrigerator (4 °C) and pipetted with the volume of approximately 5 mL to be evaluated. Terminal was opened for serial port connection and the ASCII data stream was recorded in the computer. The same experiment was repeated four times on different days. Three different oil samples were used to challenge the e-nose module. Approximately 2 mL oil was poured into a paper cup and heated to 50 °C on a hotplate. After approximately 30 s, data collection began for all ten sensor modules for the next 300 s, with data acquisition every seconds. The terminal was opened for serial port connection and the ASCII data stream recorded in the computer. The same experiment was repeated four times on different dates.

### 2.6. Data Analysis

Analysis was conducted in two steps: training and testing. For training the model, 160 sensor readings from the ADC for each oil (total of 480 data points) were imported into the Matlab^®^ classifier learner app. All 24 classifiers were trained simultaneously, and accuracy was reported as an output. The top five classifiers (fine tree, quadratic discriminant, quadratic support vector machine, cubic support vector machine, and k nearest neighbor) were selected, and their individual performance was checked by the confusion matrix and the receiver operating characteristic (ROC) curve. Once validated, each model was exported as an executable command in Matlab and challenged by a new testing dataset (360 data points; 120 each). Each classifier exported the true positive (TP), true negative (TN), false positive (FP), and false negative (FN) results. These values were used to report the statistical parameter as follows:

(1) $$\mathrm{Sensitivity}=\frac{\mathrm{TP}}{\mathrm{TP}+\mathrm{FN}}$$

(2) $$\mathrm{Specificity}=\frac{\mathrm{TN}}{\mathrm{TN}+\mathrm{FP}}$$

(3) $$\mathrm{Positive}\;\mathrm{predictive}\;\mathrm{value}=\frac{\mathrm{TP}}{\mathrm{TP}+\mathrm{FP}}$$

(4) $$\mathrm{Negative}\;\mathrm{predictive}\;\mathrm{value}=\frac{\mathrm{TN}}{\mathrm{TN}+\mathrm{FN}}$$

(5) $$\mathrm{Accuracy}=\frac{\mathrm{TP}+\mathrm{TN}}{\mathrm{TP}+\mathrm{FP}+\mathrm{TN}+\mathrm{FN}}$$

## 3. Results

### 3.1. E-Nose System

**Figure 4:**
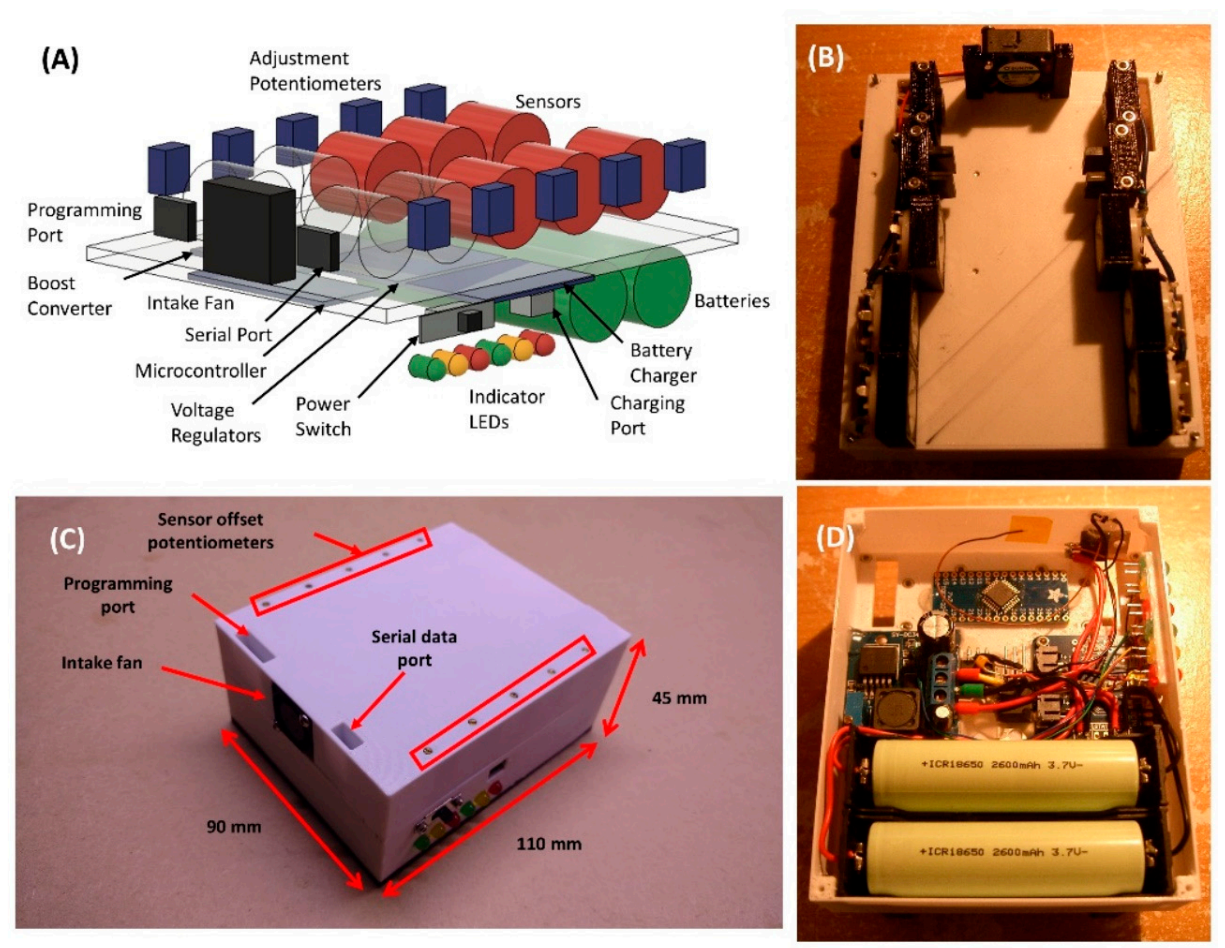
Photo of actual e-nose system. (**A**) Schematics of the functional composition of the overall system. (**B**) Inside view of top half, where each sensor is aligned to the side of the EMLA. (**C**) Photo of the fully assembled system with labels representing the control and access ports. (**D**) Inside view of the bottom half showing battery and microcontroller.

The final prototype is shown in Figure 4. Output from the sensor voltage dividers was fed directly into the microcontroller, greatly reducing the complexity and part count of this electronic nose. A fan was used to circulate the odors through the electronic nose, an arrangement much less complex than the system of pumps and valves used by most others. The compact size and light weight of this system gave it excellent portability. Because the data were sent over a standard USB serial adapter, there was less restriction regarding the type of data collection module needed.

### 3.2. Calibration Experiment

**Figure 5:**
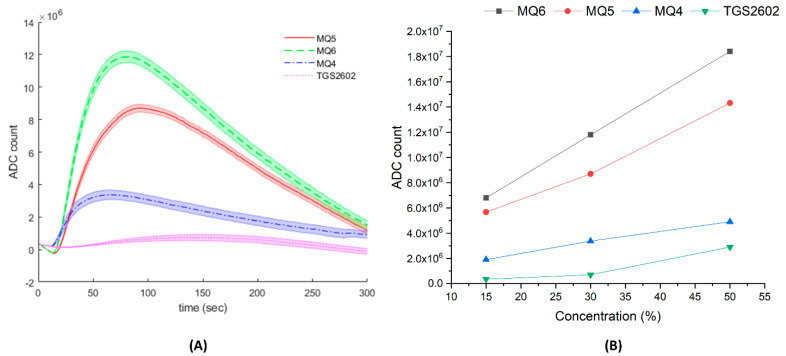
Calibration results using isopropyl alcohol. (**A**) Time-dependent sensor output for four modules (MQ4, MQ5, MQ6, and TGS2602) that showed significant response to the volatile sample of 35% isopropyl alcohol. The sample tray is positioned under the e-nose unit at time 0. (**B**) The response curve for three different concentrations. MQ6 and MQ4 show good linearity, with R2 values of 0.998 and 0.997, respectively.

The time-dependent sensor response to isopropyl alcohol concentration is shown in Figure 5. Among the 10 sensors, only four of them showed a significant response to the calibration sample (MQ4, MQ5, MQ6, TGS2602). Figure 5A shows ADC counts increasing steeply for the first minute or so after sample insertion, except for TGS2602, whose response gradually increased for 170 s. After reaching peak values, all sensor outputs gradually decreased, since the amount of diffused VOCs was limited by the droplet volume. Line elements representing the average and shaded areas show the standard deviation for the triplicate experimental samples. Figure 5B shows the correlation between the isopropyl concentration and ADC count; a good linear relationship was achieved, with R^2^ values of 0.9999 (MQ6), 0.9722 (MQ5), 0.9999 (MQ4), and 0.8945 (TGS2602), respectively.

### 3.3. Wine Experiment

**Figure 6:**
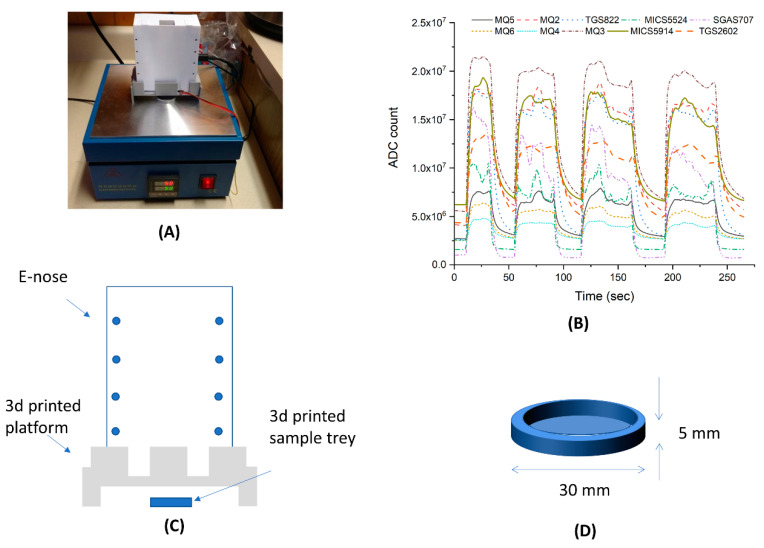
Experimental setup. (**A**) The actual setup. A heater set to 50 deg Celsius was used to control the volatility of the oil sample. (**B**) The response dynamics of the sensor for wine samples; different sensors show variations in time constants when samples were positioned in and out of the EMLA. (**C**) Schematics of the setup where sample tray, platform, enclosure for the EMLA were 3D-printed. (**D**) Actual dimensions of the disposable 3D-printed sample tray: a circle 30 mm in diameter with a 5 mm rim, printed with polylactic acid (PLA) filament.

**Figure 7:**
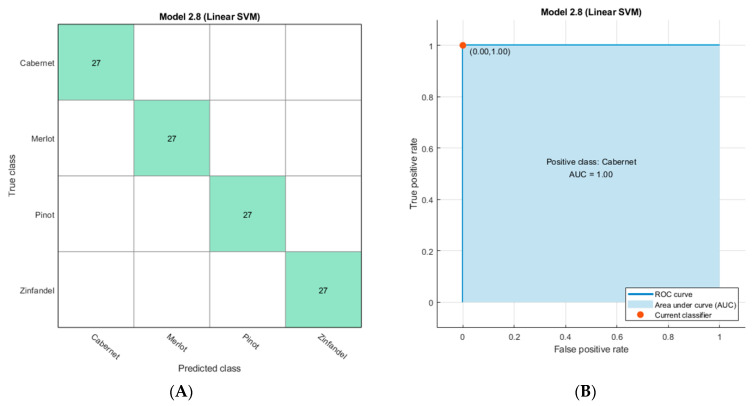
Training set results. (**A**) Confusion matrix of the training data. (**B**) ROC curve for cabernet as a positive sample by linear SVM.

Figure 6A displays the experimental setup. The EMLA unit was positioned on top of the temperature-controlled plate heater. Figure 6B shows the dynamic sensor response when a cabernet sauvignon sample was moved in and out of the interrogation position. While the sample insertion generated a quick response, removal displayed a typical first-order time response, so any subsequent sample interrogation was conducted after an interval of at least 10 min so that each sensor module was initialized. Figure 6C,D display the schematic diagram of the EMLA unit, 3D-printed platform, and sample holders. Figure S2 displays the survey of the classifiers conducted on the Matlab classifier learner while Figure S3 shows an example of training model from linear SVM. Using the test-set data, all 24 classifiers were challenged, and their cross-validation results tabulated. Here, accuracy was plotted against all classifiers and the six best performers were selected for the testing phase. The performance of the trained model is shown in Figure 7 via the cross-validation matrix and ROC curve. Table 1 shows the testing results for classifiers challenged with a new dataset of wine samples; statistical results were reported using Equations (1)–(5). Among the models tested, both linear and quadratic SVM classifiers provided the best performance, including sensitivity and specificity.

Figure S2 shows the representative scatter plot of sensor readings for a sensor combination (TGS822 and MQ2). As expected, some sensors showed overlapping sensitivities to secondary chemical species, so these results were displayed to provide the group separation by different wine types.

### 3.4. Oil Experiment

The EMLA unit was positioned on top of the plate heater and the temperature was set to 50 deg Celsius to enhance the amount of volatile organic compounds available to the sensor. As with the wine sample, training sets were captured, and several models were trained (Figure S2). The top five classifier models (fine tree, quadratic discriminant, quadratic SVM, cubic SVM, and fine KNN) among the 24 tested were challenged by the testing set. The performance of the training set is shown in Figure S4. The overall results for the testing set varied widely. Except for the quadratic SVM classifier, the methods showed a sensitivity or specificity of less than 50% for an oil sample. Overall results are summarized in Table 2.

## 4. Discussion

In the field of portable and field-deployable detection systems, smartphone-based systems have been recently highlighted, for the most part focusing on optical transduction [27,28,29]. However, for odor detection, metal-oxide sensors provide better options in terms of sensor availability and simple transduction of volatile organic compounds into voltage signals. The proposed e-nose design was implemented based on the limited choice of available metal-oxide chemical sensors. While cost reduction and miniaturization were possible by employing this type of sensor, there were a few limitations as well. First, as shown in Table S1, many sensors provide a primary response to certain chemical species along with a large number of secondary (weak) responses that overlap among various sensor types and vendors. This makes the deterministic approach less effective, since EMLA will not generate a unique response when a single species of chemical is present. However, the relative sensor response was not directly comparable among different vendors, so a statistical learning method was employed to teach the sensors to acquire the characteristics of each sample under investigation.

While the calibration experiment was performed observing the sensitivity and dynamics from a single chemical species, some sensor dynamics could be explored. Based on the calibration experiment with isopropyl alcohol, the sensors MQ6 (R^2^ = 0.9999), MQ5 (R^2^ = 0.9722), MQ4 (R^2^ = 0.9999), and TGS2602 (R^2^ = 0.8945) resulted in a linear relationship between concentration and ADC count. This means that the EMLA unit has potential for measuring the concentration of compounds and mixtures of foods with quantitation. One of the second characteristics observed in using the metal-oxide sensor module was that the sensors have different response times and cooling times. Based on Figure 6, all the sensors responded with a typical first-order system response, having time constants of 30–40 s, depending on the sensor type. However, one sensor module (SGAS 707) showed an extremely slow response and cool down; for future system development, this module will be replaced by another with similar characteristics and a faster response time. As noted from other reviews, metal-oxide-type sensors’ reactions can be affected by local temperature and humidity levels [30,31]. One of the limitations of the proposed device is the passive control of the environment. Therefore, signal fluctuation could be generated from the passive nature of the temperature and humidity control.

For the circuit readout, oversampling was used to improve the effective resolution of the ADC. This allows for the internal ADC to be used, reducing the part count of the e-nose. However, oversampling came at the cost of some extra processing and a reliance on uncorrelated noise in order for oversampling to work properly.

The use of a fan instead of pumps and valves allowed for a simpler system compared to other electronic noses. However, this means that the electronic nose cannot precisely control the sampling conditions. Despite this drawback, the electronic nose was able to successfully distinguish between different brands and types of wine and oil samples. Matlab’s classifier learner app provided a quick and broad spectrum of the classification models. A total of 24 linear classifiers can be directly implemented, with the additional help of principal component analysis to reduce the dimensionality. The current EMLA system relies on Matlab-based offline analysis of the captured data. Final implementation of EMLA as a field-deployable unit will require porting the trained model into the micro-processor unit so that on-board analysis can be conducted.

## 5. Conclusions

A metal-oxide sensor-based electronic nose system called EMLA was presented. This system utilizes ten metal-oxide sensors that were selected based on their availability and the scope of their response to chemical species. A portable unit was designed to be battery-operated and a fan was used to control the flow rate of the sample inlet. A Matlab-based machine learning algorithm was implemented. The best results overall were achieved by a quadratic SVM classifier, with a minimum classification accuracy of 97% for four wine samples and 93% for three oil samples.

## Figures and Tables

**Table 1:** Testing results from six different classifier models for wine samples.

Classifier	Type	Sensitivity	Specificity	PPV	NPV	Accuracy
Linear discriminant	Zinfandel	1	1	1	1	1
	Cabernet sauvignon	1	1	1	1	1
	Pinot noir	1	0.8889	1	0.9643	0.9722
	Merlot	0.963	1	0.9	1	0.9722
Quadratic discriminant	Zinfandel	1	1	1	1	1
	Cabernet sauvignon	1	1	1	1	1
	Pinot noir	1	0.5	1	0.8571	0.875
	Merlot	0.833	1	0.6667	1	0.875
Linear SVM	Zinfandel	1	1	1	1	1
	Cabernet sauvignon	1	1	1	1	1
	Pinot noir	1	0.944	1	0.9818	0.9861
	Merlot	0.9815	1	0.9474	1	0.9861
Quadratic SVM	Zinfandel	1	1	1	1	1
	Cabernet sauvignon	1	1	1	1	1
	Pinot noir	1	0.944	1	0.9818	0.9861
	Merlot	0.9815	1	0.9474	1	0.9861
Bayes Gaussian	Zinfandel	1	1	1	1	1
	Cabernet sauvignon	1	0.5	1	0.8571	0.875
	Pinot noir	1	0.833	1	0.9474	0.9583
	Merlot	0.7778	1	0.6	1	0.8333
KNN fine	Zinfandel	1	1	1	1	1
	Cabernet sauvignon	1	0.5	1	0.8571	0.875
	Pinot noir	1	0.9444	1	0.9818	0.9861
	Merlot	0.8148	1	0.6429	1	0.8611

**Table 2:** Testing results using six different classifier models for oil samples.

Classifier.	Type	Sensitivity	Specificity	PPV	NPV	Accuracy
Quadratic discriminant	Grapeseed oil	1	0.2	1	0.7143	0.7333
	Peanut oil	0.115	0.95	0.3493	0.8214	0.3933
	Olive oil	0.96	0	0	0.6575	0.64
Quadratic SVM	Grapeseed oil	1	1	1	1	1
	Peanut oil	0.945	0.91	0.8922	0.9545	0.9333
	Olive oil	0.955	0.89	0.9082	0.9455	0.9333
Cubic SVM	Grapeseed oil	1	1	1	1	1
	Peanut oil	0.855	0.4	0.5797	0.7403	0.7033
	Olive oil	0.7	0.71	0.542	0.8284	0.7033
Fine Tree	Grapeseed oil	1	1	1	1	1
	Peanut oil	0.57	0.86	0.5	0.8906	0.6667
	Olive oil	0.93	0.14	0.5	0.6838	0.6667
KNN fine	Grapeseed oil	1	0.89	1	0.9479	0.9633
	Peanut oil	0.86	0.54	0.6585	0.789	0.7533
	Olive oil	0.715	0.72	0.5581	0.8363	0.7167

## Data Availability

The datasets generated from the current study are available from the corresponding author on reasonable request.

## Supplementary Materials

The following are available online at https://www.mdpi.com/article/10.3390/s21113923/s1. Table S1. List of candidate sensors. Bold face sensors were selected to be used in the EMLA unit (10 different sensors). They were selected based on the wide variety of primary responses to the chemical species. Figure S1: Full electronic CAD diagram for the EMLA unit. Four major components were microcontroller unit, sensors unit, heater and fan control unit, and power switch unit. Figure S2: Survey of accuracy for 24 different classification methods. Tree (6 types), discriminant analysis (2), Baysian (2), SVM (6), and KNN (7). (A) Wine sample (B) Oil sample. Figure S3: Scatterplot of four different wine samples depicted by two sensors: TGS822 and MQ2. Figure S4: (A) Confusion matrix and (B) area under the curve graph for classification of wine samples.
