# Supplementary material for: Design and Validation of a Portable Machine Learning-Based Electronic Nose
Source: Sensors (Basel). 2021 Jun 7;21(11):3923. doi: 10.3390/s21113923 (PMC8201040; doi:10.3390/s21113923)
Supplement: Supplementary file 1 [file sensors-21-03923-s001.zip › sensors-1231823-supplementary.pdf]

Design and Validation of a Portable Machine Learning-Based Electronic Nose

Yixu Huang [1], Iyll-Joon Doh [1], and Euiwon Bae [1,*]

[1]*Applied Optics Laboratory, School of Mechanical Engineering, Purdue University, West Lafayette, Indiana 47907, USA*

**Corresponding author: ebae@purdue.edu*

Table S1. List of candidate sensors. Bold face sensors were selected to be used in the EMLA unit (10 different sensors). They were selected based on the wide variety of primary responses to the chemical species.

Sensor	Manufacturer	Cost /Unit	Primary Response	Secondary Response
**MQ-6**	Figaro	$4.95	C_3H_8	CH_4, C_2H_5OH
**MQ-4**	Figaro	$4.95	CH_4,	C_3H_8, C_2H_5OH
**MQ-3**	Figaro	$4.99	C_2H_5OH	C_4H_{10}, CH_4, CO
MQ-7	Figaro	$5.99	H_2	CH_4, C_2H_5OH, CO
MQ-8	Figaro	$7.95	H_2	CH_4, C_2H_5OH, CO
**MQ-5**	Figaro	$6.99	C_4H_{10}	CH_4, C_2H_5OH, H_2
**MQ-2**	Figaro	$7.99	C_3H_8	C_3H_8, CH_4, C_2H_5OH, H_2
SGAS701	Renesas	$31.24	H_2	C_7H_8, C_3H_6O, C_8H_{18}, C_4H_8
**SGAS707**	Renesas	$31.24	C_2H_5OH	C_7H_8, C_3H_6O, C_8H_{18}, C_4H_8
SGAS711	Renesas	$31.24	H_2	C_3H_8, CH_4
**MICS-5914**	SGX sensor	$9.95	NH_3	C_2H_5OH, C_3H_8, C_4H_{10}
**MICS-5524**	SGX sensor	$8.91	CO	C_2H_5OH, H_2, C_4H_{10}
TGS2600-B00	Figaro	$20.79	H_2	C_2H_5OH, C_4H_{10}
TGS2610-C00	Figaro	$25.50	C_3H_8	CH_4, C_4H_{10}, C_2H_5OH
TGS2620-C00	Figaro	$25.50	C_2H_5OH	H_2, C_4H_{10}
TGS813-A00	Figaro	$25.50	H_2	C_2H_5OH, C_4H_{10}, CH_4
**TGS2602-B00**	Figaro	$25.50	C_7H_8	C_2H_5OH
TGS2611-C00	Figaro	$25.50	CH_4	C_4H_{10}, C_2H_5OH
**TGS822-A00**	Figaro	$25.50	C_3H_6O	C_2H_5OH, C_6H_6
TGS6810-D0	Figaro	$28.84	C_4H_{10}	C_3H_8, CH_4

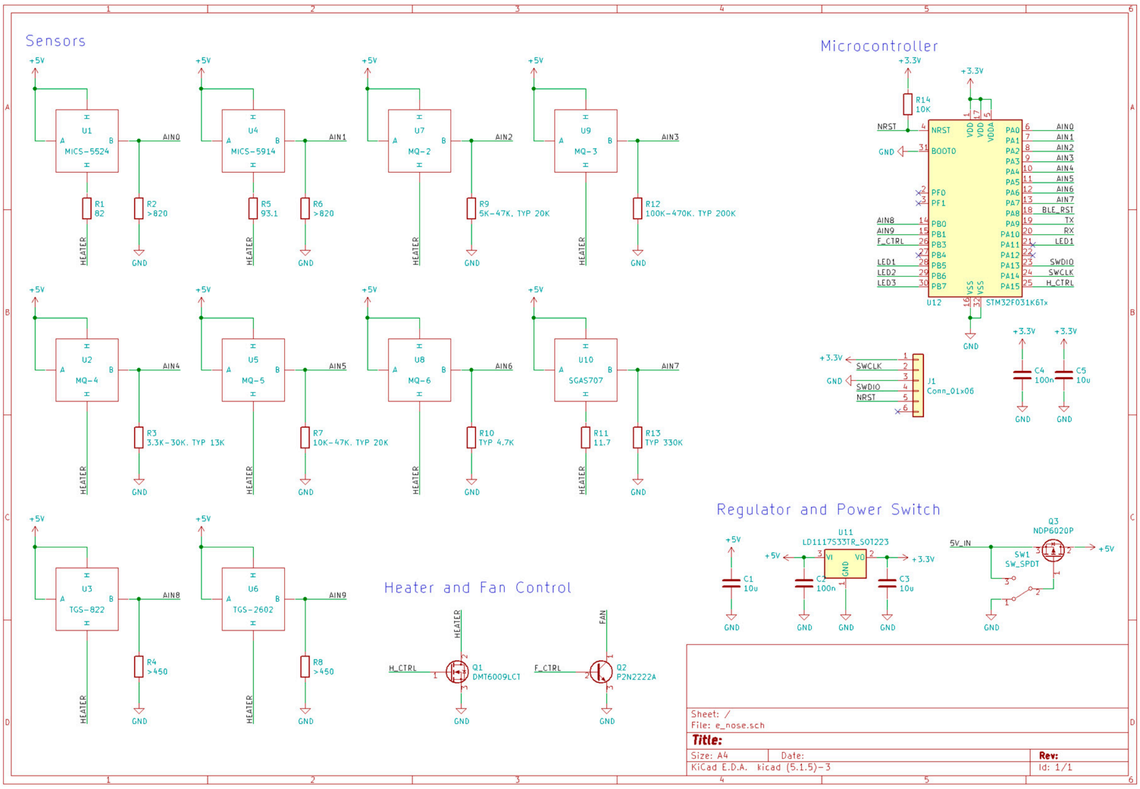

Figure S1. Full electronic CAD diagram for the EMLA unit. Four major components were microcontroller unit, sensors unit, heater and fan control unit, and power switch unit.

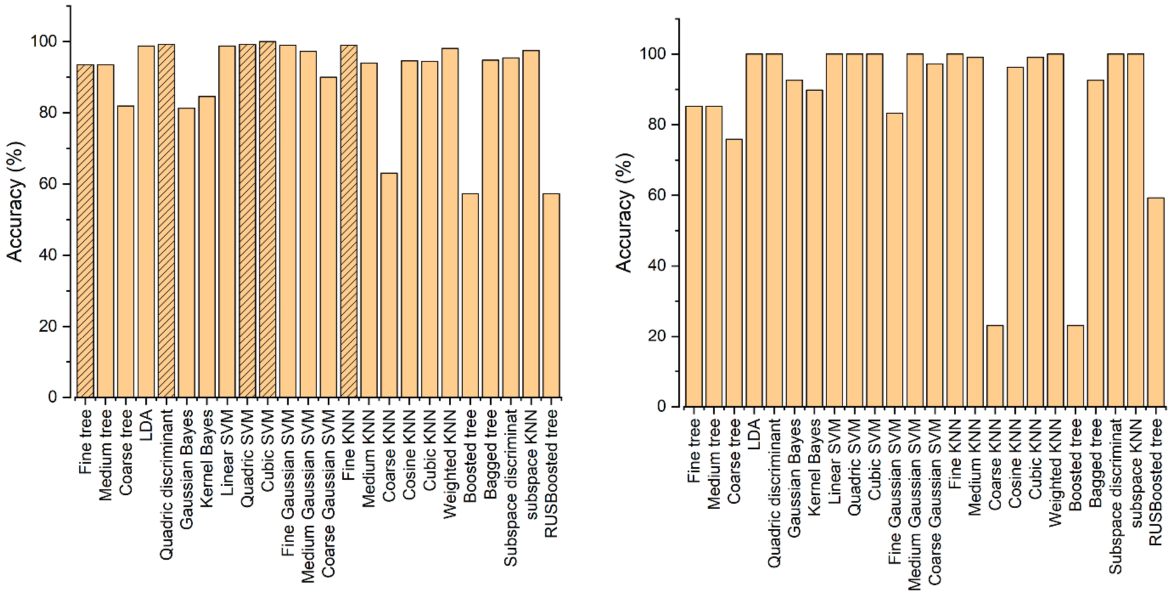

(A)   (B)

Figure S2. Survey of accuracy for 24 different classification methods. Tree (6 types), discriminant analysis (2), Baysian (2), SVM (6), and KNN (7). (**A**) Wine sample (**B**) Oil sample.

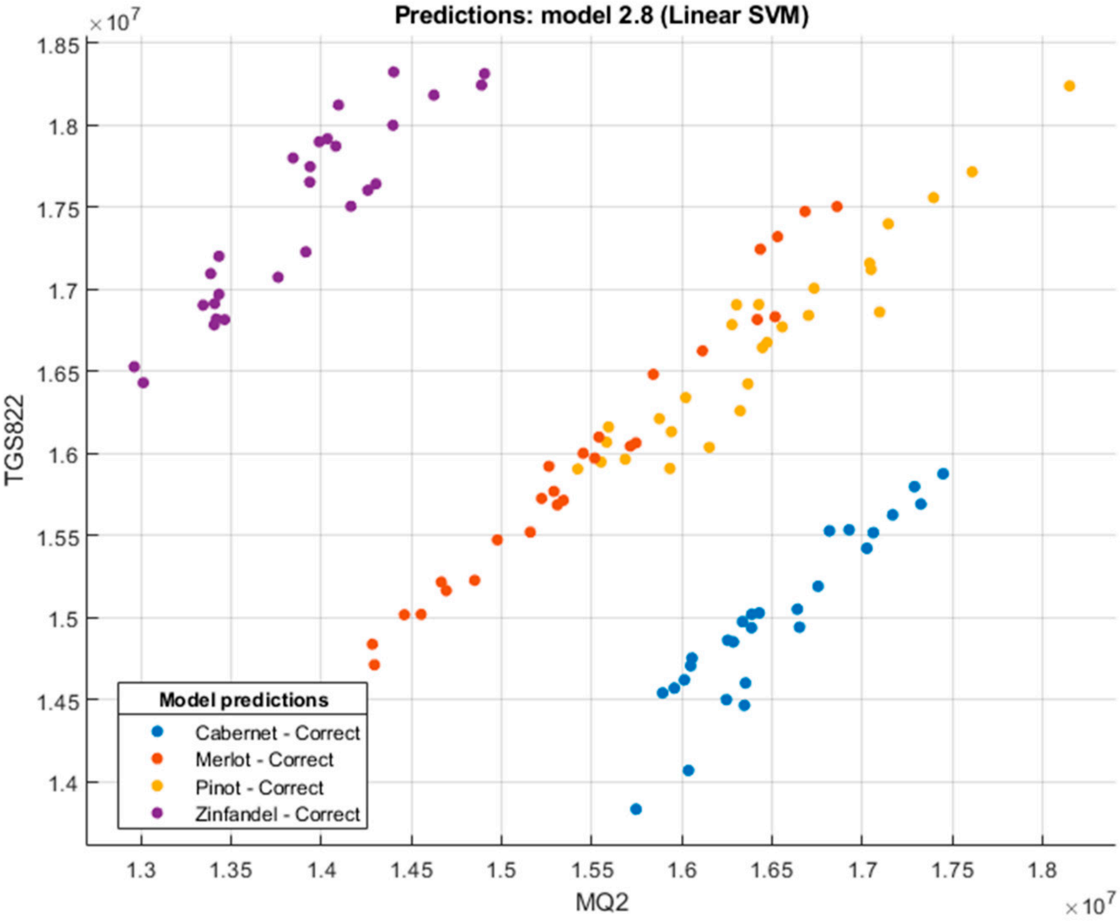

Figure S3. Scatterplot of four different wine samples depicted by two sensors: TGS822 and MQ2.

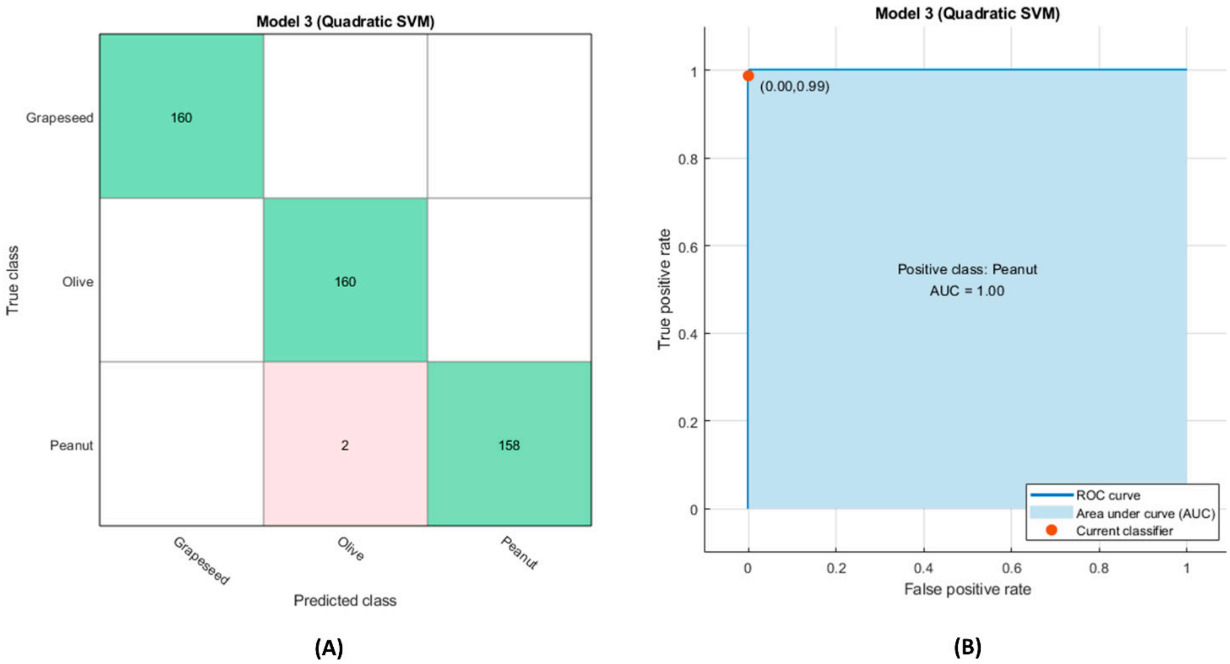

Figure S4. (**A**) Confusion matrix and (**B**) area under the curve graph for classification of wine samples.
